# Mutation of N-glycosylation Sites in Salmonid Alphavirus (SAV) Envelope Proteins Attenuate the Virus in Cell Culture

**DOI:** 10.3390/v12101071

**Published:** 2020-09-24

**Authors:** Ida Aksnes, Turhan Markussen, Stine Braaen, Espen Rimstad

**Affiliations:** Department of Paraclinical Sciences, Norwegian University of Life Sciences, 0454 Oslo, Norway; ida.aksnes@nmbu.no (I.A.); turhan.markussen@nmbu.no (T.M.); stine.braaen@nmbu.no (S.B.)

**Keywords:** k salmonid alphavirus, Pancreas disease, reverse genetics, virulence, attenuation, N-glycosylation

## Abstract

Salmonid alphavirus (SAV) is the cause of pancreas disease and sleeping disease in farmed salmonid fish in Europe. The spread of these diseases has been difficult to control with biosecurity and current vaccination strategies, and increased understanding of the viral pathogenesis could be beneficial for the development of novel vaccine strategies. N-glycosylation of viral envelope proteins may be crucial for viral virulence and a possible target for its purposed attenuation. In this study, we mutated the N-glycosylation consensus motifs of the E1 and E2 glycoproteins of a SAV3 infectious clone using site-directed mutagenesis. Mutation of the glycosylation motif in E1 gave a complete inactivation of the virus as no viral replication could be detected in cell culture and infectious particles could not be rescued. In contrast, infectious virus particles could be recovered from the SAV3 E2 mutants (E2319Q, E2319A), but not if they were accompanied by lack of N-glycosylation in E1. Compared to the non-mutated infectious clone, the SAV3-E2319Q and SAV3-E2319A recombinant viruses produced less cytopathic effects in cell culture and lower amounts of infectious viral particles. In conclusion, the substitution in the N-linked glycosylation site in E2 attenuated SAV3 in cell culture. The findings could be useful for immunization strategies using live attenuated vaccines and testing in fish will be desirable to study the clone’s properties in vivo.

## 1. Introduction

Salmonid alphavirus (SAV), formally named *Salmon pancreas disease virus* (SPDV), is the causative agent of pancreas disease (PD) in Atlantic salmon (*Salmo salar*) and sleeping disease (SD) in rainbow trout (*Oncorhyncus mykiss*) [1]. The diseases are widespread in salmonid aquaculture in Europe and may cause high fish mortality and reduced weight gain resulting in great economic losses for the industry. Vaccines based on an inactivated virus were used in farmed salmonids for some years, and has been shown to reduce virus shedding, as well as the severity and mortality of the disease [2]. However, the number of PD outbreaks registered have not declined, and in Norway, 152 outbreaks were reported in 2019 [3]. Recently, a DNA vaccine against PD was approved for use as the first DNA vaccine in the EU. Fresh and sea water trials indicated reduced mortality and less damage to pancreas, heart and muscle tissue in the vaccinated fish [4] but did not block SAV infection.

There are six phylogenetically distinct subtypes of SAV (SAV1–6) that for the most part are linked to geographical location. SAV1 is common in Ireland and Scotland. SAV2 is found in freshwater-reared rainbow trout in continental Europe [5] as well as in salmonids in sea cages in England, Scotland and Norway [6]. SAV3 is common in Norway [7], and SAV4, 5 and 6 are occasionally detected in the British Isles [8].

SAV is a member of the genus *Alphavirus*, family *Togaviridae*. Alphaviruses infect a wide range of vertebrate animals and mainly cause arthritic diseases and encephalitis. Alphaviruses found in mammals are arthropod-borne and replicate in both the invertebrate vectors and the vertebrate host. SAV is an exception to this because it apparently lacks an invertebrate vector and transmits horizontally between individuals.

Alphaviruses derive their envelope from the host cell membrane and the viral glycoproteins embedded in the envelope form 80 spikes [9]. The viral genome is a positive-sense single-stranded RNA with two open reading frames (ORFs). The ORF1 encodes the four non-structural proteins (nsP1–nsP4), and the ORF2 encodes the five structural proteins; the capsid, E3, E2, 6K and E1. The junction region between ORF1 and ORF2 is a promoter that initiates the transcription of a subgenomic mRNA that translates into a precursor polyprotein containing the structural protein sequences [10]. The capsid protein is autocatalytically cleaved off and the remaining part of the polyprotein, p62–6K-E1, translocates to the endoplasmic reticulum for further processing, which includes a heterodimer formation of p62 and E1. This is followed by the oligomerization of three heterodimers that will, following transport to the plasma membrane through the host secretory system, form the viral spikes. P62 is cleaved into E3 and E2 in the trans-Golgi network by a cellular enzyme [11]. For SAV, the optimal temperature for replication in cell culture is 10–15 °C, and virions are not formed at temperatures above 18 °C. The critical determinant for this temperature-dependent SAV virion formation is the translocation and presentation of the E2 glycoprotein at the cell surface. The structural proteins, E1 and E2, form a heterodimer that assembles in a trimer that constitutes the viral spikes [11]. During viral entry, E2 is responsible for receptor binding, whereas E1 mediates the fusion of viral and cellular membranes [12].

The E1 and E2 proteins are covalently modified by oligosaccharide chains. The glycosylation of viral envelope proteins allows for proper folding and intracellular trafficking, which facilitates efficient virion production and release. N-linked glycosylation is the most common form of glycosylation in viral proteins, where a high mannose core is attached to an amide nitrogen of Asn within the conserved Asn-X-Ser/Thr motif [13,14,15]. The glycosylation of viral proteins increases structural diversity and function, and may impact receptor recognition, viral infectivity and alter recognition by host immune cells [16]. The type of glycan attached to viral envelope proteins depends on the host cell glycosylation machinery, and the variation in the extent and patterns of glycans can be species specific [17,18].

The N-linked glycosylation sites of E1 and E2 are mostly conserved for alphaviruses, despite different hosts and different modes of transmission, suggesting they are linked to important biological properties of the virus [11]. Functional studies of the role of N-linked glycosylation have been performed for several alphaviruses. For Ross river virus (RRV), a lack of an E1 glycosylation site resulted in attenuation with improved viral clearance in infected mice, whereas RRV lacking a glycosylation site in E2 showed significantly reduced replication efficiency in the mosquito vector [19]. Similarly, Sindbis virus (SINV) lacking N-glycosylation in E1 had reduced virulence and impaired replication in both mosquito and vertebrate cells, and a lack of glycosylation in both E1 and E2 reduced virulence further. Interestingly, the lack of glycosylation in SINV E2 only increased the replication and virulence in inoculated mice due to the increased efficiency of binding to heparin sulphate and facilitated viral entry into cells [20,21]. Mutations targeting viral glycans can therefore be a strategy in vaccine development. Attenuated vaccines create a strong and lasting immune response and could be beneficial for the control of SAV-induced diseases if risk analysis indicates that the safety of using live virus vaccines in aquaculture is high.

The functional roles of the SAV E1 and E2 N-glycosylation sites have not yet been studied. We generated a panel of SAV3 mutant viruses lacking these sites in E1 and/or E2 and compared them to a non-mutated clone and a virus isolate. Experimental infections were performed in salmonid cell lines where viral RNA replication, the induction of cytopathic effect (cpe), and the production of infective virus particles were investigated.

## 2. Materials and Methods

### 2.1. Computer Analyses of SAV3 E1 and E2 Sequences

The SAV3 E1 and E2 proteins sequences were analyzed in silico for the prediction of N-glycosylation The SAV3 E1 and E2 proteins sequences were analyzed in silico for the prediction of N-glycosylation sites. Multiple sequence alignments of E1 and E2 protein sequences from a SAV3 infectious clone (see below), Venezuelan equine encephalitis virus (VEEV), Eastern equine encephalitis virus (EEEV), Chikungunya virus (CHIKV), RRV, Semliki Forest virus (SFV) and SINV were performed using MUSCLE [22] (available from https://www.ebi.ac.uk/Tools/msa/muscle/m), MEGAX [23] (available at www.megasoftware.net) and AlignX software (Vector NTI Advance^TM^ 11, Invitrogen, Carlsbad, CA, USA). Protein sequence alignments were further processed in Jalview 2.11.0 for display purposes [24] (downloaded from https://www.jalview.org/). Pairwise amino acid sequence identities and similarities were calculated using the SIAS server (http://imed.med.ucm.es/Tools/sias.html). Protein secondary structure predictions were performed using PSIPRED 4.0 (http://bioinf.cs.ucl.ac.uk/psipred/) [25], and the localization of N-linked glycosylation sites in the two SAV proteins predicted using the NetNGlyc 1.0 server (available at http://www.cbs.dtu.dk/services/NetNGlyc/). In order to spatially visualize the localization of the predicted E1 and E2 N-glycosylation sites in SAV3, structure homology modelling was performed on both proteins using the threading method provided by the I-TASSER server, available at https://zhanglab.ccmb.med.umich.edu/I-TASSER/ [26,27]. The accuracy of the 3D structure models generated can be evaluated from the accompanying TM and C-scores. The TM-score measures the global fold similarity of protein structures, and the magnitude of the score for random structure pairs is length-independent. The TM-score range is 0–1, where 1 represents a perfect match. In general, scores higher than 0.5 assume roughly the same fold. The C-score is a confidence score for estimating the quality of the predicted models by I-TASSER, calculated based on the significance of threading template alignments and the convergence parameters of the structure assembly simulations [26,27]. Structures were visualized and analysed in Swiss-PdbViewer 4.1.0 [28].

### 2.2. Cell Cultures

Chinook salmon embryo cells (CHSE-214) (RRID:CVCL_278) and Chum salmon heart-1 cells (CHH-1) (RRID:CVCL_4143) were used. Both cell lines were cultivated at 20 °C in a grow out medium, Leibovitz (L15) supplemented with 10% heat inactivated fetal bovine serum (FBS), 2-mercaptoethanol (40 µM) and gentamicin–sulphate (50 µg/mL) (all from Life technologies, Paisley, Scotland, UK), or in maintenance medium which contained 2% FBS.

### 2.3. Plasmid Constructs

A SAV3 cDNA infectious clone (prSAV) [29] was used as a template for the construction of the mutated virus clones. An XbaI site was introduced in the junction area of the genome of rSAV3, as a tag to separate the progeny virus derived from the rSAV3 infectious clone from that of wild-type SAV3. Single-site substitutions were introduced using QuickChange site-directed mutagenesis (Agilent, Santa Clara, CA, USA) following the manufacturer’s instructions. Primers containing the desired nucleotide substitutions were designed using the QuickChange T_m_ calculator, https://www.agilent.com/store/primerDesignProgram.jsp. Six different SAV3 cDNA clones were made with mutations in their E1_35_ and/or E2_319_ N-glycosylation sites, where the Asn (N) in the Asn-X-Ser/Thr motif were substituted with either a Gln (Q) or Ala (A), (Table 1). The design and production of mutated SAV were performed in approved laboratory facilities at the Norwegian University of Life Sciences.

### 2.4. Transfection

In each transfection experiment, a 0.5 µg endotoxin-free plasmid and 100 µL Ingenio solution (Mirus Bio LLC, Madison, WI USA) were added to 4 × 10^6^ CHSE-214 cells. The cells were transfected using the Amaxa Nucleofector device (Lonza, Basel, Switzerland). Transfected cells were transferred to either 25 cm^2^ flasks (Corning, NY, USA) or 24-well plates and incubated at 20 °C for 24 h before being transferred to 15 °C. Cells in flasks were incubated for 14 days (passage 0, P0). Cells growing in 24-well plates were used for indirect fluorescent antibody test (IFAT). These cells were incubated for four days.

### 2.5. Indirect Fluorescent Antibody Technique

Transfected CHSE-214 were washed in Dulbecco’s PBS (DPBS) containing sodium azide. Cells were further fixed and washed using the Intracellular Fixation & Permeabilization Buffer Set (eBioscience, San Diego, CA, USA). Following the second wash, the cells were incubated with a monoclonal antibody against E2 (mouse anti-E2 17H23) (1:1000) [30] for 60 min at room temperature, washed twice with permeabilization buffer and incubated for 1 h at room temperature with Alexa Fluor 488-conjugated anti-mouse antibody (1:400) (Molecular Probes, Life Technologies, Eugene, OR, USA). Nuclear DNA was stained with Hoechst 33,342 (ThermoFisher, Waltham, MA USA). Non-transfected cells and cells without added primary antibodies were used as negative controls. Cells were examined under an inverted Olympus IX81 fluorescence microscope.

### 2.6. Recovery and Passage of Mutated Clones

Fourteen days after transfection, 1 mL of supernatant from transfected CHSE-214 cells were transferred to CHH-1 cells (Passage one, P1) in 25 cm^2^ flasks (Corning **^®^**). We used CHH-1 cells, originally from Chum salmon, for the comparative assessment of the effects of putative deglycosylation of SAV3 E2 on the development of cpe, viral RNA replication and the production of the infectious virus. The inoculated CHH-1 cells were kept one hour at 15 °C, before 5 mL maintenance media were added followed by 14 days at 15 °C. Four serial passages of supernatant were performed (P1–P4). The virus endpoint dilution assay and RT-qPCR (see below) were performed to quantify the infectious viral particles and RNA, respectively, in the cell-culture media. To ensure that the E2_319Q_ and E2_319A_ mutations were still intact after the final passage (P4), Sanger sequencing (GATC Biotech AG, Konstanz, Germany) of the E2 coding region were carried out.

### 2.7. Replication of Viral RNA

Two parallel wells with 4 × 10^5^ CHH-1 cells were infected with either rSAV3, rSAV3 E2_319Q_ or rSAV3 E2_319A_ at a titre of 10^4^ tissue culture infective dose (TCID_50_/mL). In short, the cells were washed twice with PBS before 1 mL of virus suspension was added and then incubated for one hour at 15 °C, followed by the addition of 4 mL of maintenance medium. The cell culture medium and cells were sampled at 2, 6, 12 and 24 h post-infection (hpi), and 3, 7, 9 and 14 days post infection (dpi). At each sampling, the cells were examined by phase contrast microscopy. The supernatant was centrifuged at 1200 rpm at 4 °C for 5 min, and 250 µL was added to 750 µL Trizol LS (InvitrogenTM, Carlsbad, CA, USA). The cells were washed twice with PBS followed by cell lysis after adding 1 mL Qiazol lysis reagent (Qiagen, Hilden, Germany) per well. Both the remaining supernatant and cells were then stored at −80 °C. The end-point titration of the supernatant at 24 hpi, 3, 7, 9 and 14 dpi was performed.

### 2.8. Analysis of Virus Titers

Infectious virus was titrated in CHH-1 cells. Serial 10-fold dilutions of the supernatant from infected cells, P4, at 24 hpi, 3, 7, 9, 14 dpi were added in eight parallels into a 96-well plate containing a confluent cell monolayer. The plates were incubated for ten days before being assessed for cpe, and in addition, IFAT with anti-E2 was done. The Spearman–Kärber algorithm was used for the calculation of virus titers.

### 2.9. RNA Isolation and RT-qPCR

Total RNA from P0 to P4 was isolated from 250 µL supernatant by the use of TRIzol LS as recommended by the manufacturer (Invitrogen, Carlsbad, CA, USA). Following the addition of chloroform and centrifugation, the aqueous phase was transferred to a RNeasy mini spin column. The remaining RNA purification procedure followed the instructions of this manufacturer (Qiagen, Hilden, Germany). RNA was eluted in 50 µL RNase free water. cDNA was synthesized using the QuantiTect ^®^ Reverse Transcription kit (Qiagen). For each sample, two reaction mixtures were made, with or without the reverse transcriptase enzyme. QPCR was performed using 3 µL cDNA in a total reaction volume of 13 µL. The primers and probe targeted the SAV nsP1 coding sequence [31]. Reactions were made using 400 nM primer, 300 nM probe, 6.5 µL TaqMan^®^ Gene Expression Master Mix and 2.3 µL RNase-free water. The cycling parameters were 50 °C/2 min and 95 °C/15 min, followed by 40 cycles of 95 °C/15 s and 60 °C/1 min, using an AriaMx real-time PCR system (Agilent, Santa Clara, CA, USA).

Identical procedures for RNA isolation, cDNA synthesis and RT-qPCR were performed for the infected cells, except omitting the enzyme as a negative control during reverse transcription. The total RNA concentration of infected cells was determined by spectrophotometry using the Nanodrop ND1000 (Nanodrop Technologies, Wilmington, DE, USA). cDNA was synthesized from 750 ng RNA using the Quantitect**^®^** Reverse Transcription kit (Qiagen) containing gDNA wipe out buffer, following the manufacturer’s instructions. qPCR was performed using 15 ng cDNA in each reaction.

## 3. Results

### 3.1. Prediction of N-glycosylation Sites in SAV3 E1 and E2

**Figure 1:**
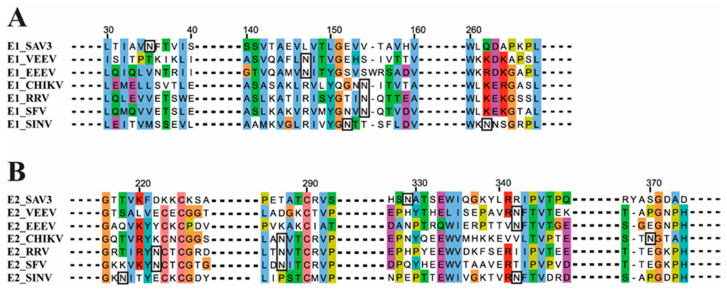
Location of the N-glycosylation sites in SAV3 E1 and E2. Multiple sequence alignment of (**A**) SAV3 E1 and (**B**) SAV3 E2 with the corresponding protein sequences from a selection of other alphaviruses. Predicted N-glycosylation sites are shown in black boxes. Except for SAV3, the sites of N-glycosylation in E1 and E2 have been verified by structural studies [32,33,34,35,36,37,38,39,40]. VEEV = Venezuelan equine encephalitis virus (P09592), EEEV = Eastern equine encephalitis virus (ANB41743), CHIKV = Chikungunya virus (AEA10291), RRV = Ross River virus (P08491), SFV = Semliki Forest virus (NP_819008, NP_819006) and SINV = Sindbis virus (CAA24684). Dotted lines in bold indicate the sequence sections removed for display purposes.

One N-glycosylation site was predicted in both SAV3 E1 (E1_35N_) and SAV3 E2 (E2_319N_). The location of these sites in SAV3 compared to that of other alphaviruses are shown in Figure 1. The alignments of the complete E1 and E2 sequences are shown in Figure S1.

**Figure 2:**
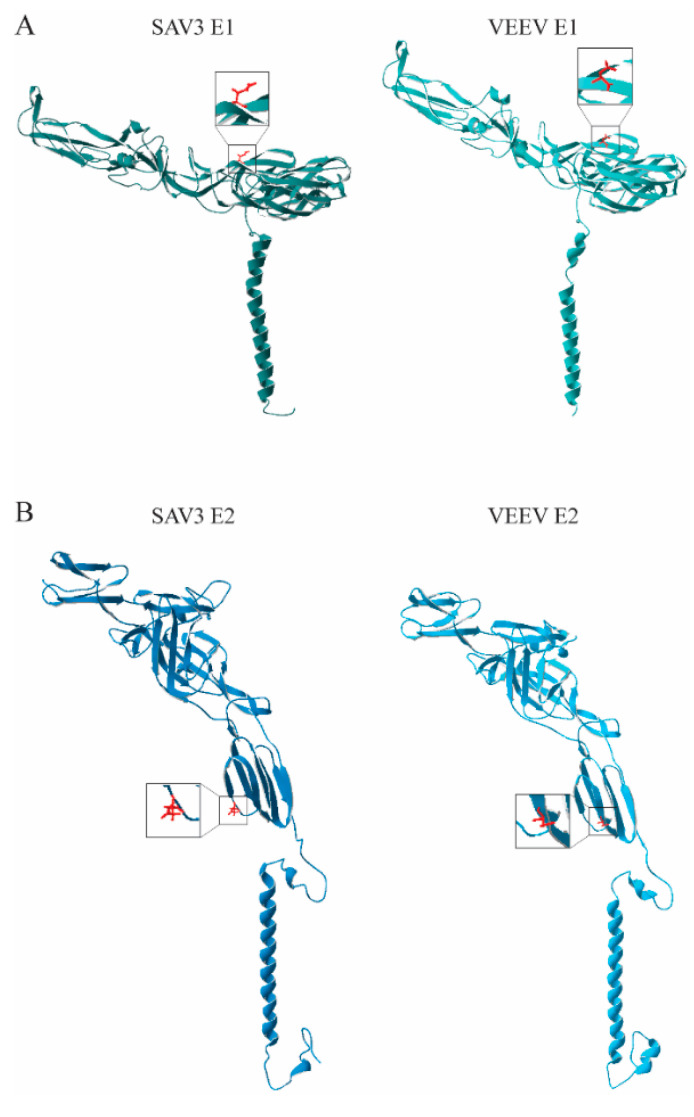
Ribbon diagrams of the modeled SAV3: (**A**) E1 and (**B**) E2 proteins compared with that of the best fitting templates, VEEV E1 and E2 (PDB IDs: 3J0C_A, 3J0C_B), as predicted by I-TASSER. Asparagine side chains on SAV3 E1 (N_35_) and E2 (N_319_) were predicted to be involved in N-linked glycosylation, and the corresponding sites on VEEV E1 (N_134_) and VEEV E2 (N_318_), are shown in red.

The pairwise amino acid sequence identities and similarities between SAV3 E1 to that of other alphaviruses were 38.2–40% and 49.3–50.8%, respectively. For SAV3 E2, the corresponding numbers were 25.8–29.5% and 37.4–42.0% (Tables S1 and S2). However, the secondary structure predictions suggested a high level of structural conservation for both proteins (Figure S2). In order to spatially visualize the localization of the SAV3 N-glycosylation sites, structure homology modeling was performed for the SAV3 E1 and E2 proteins using the I-TASSER server (Figure 2). The server identified the E1 and E2 from VEEV as the most appropriate templates for the modeling, with TM scores of 0.94 ± 0.05 (E1) and 0.99 ± 0.04 (E2) and C-scores of 1.6 (E1) and 2.00 (E2). The scores suggest a higher level of secondary structure conservation for E2 than E1. At the primary sequence level, on the other hand, the opposite was observed as pairwise sequence comparisons indicated that the E2 protein sequences were roughly 10% less conserved between the two alphaviruses than E1 (Tables S1 and S2).

### 3.2. Recovery of Recombinant Viruses

**Figure 3:**
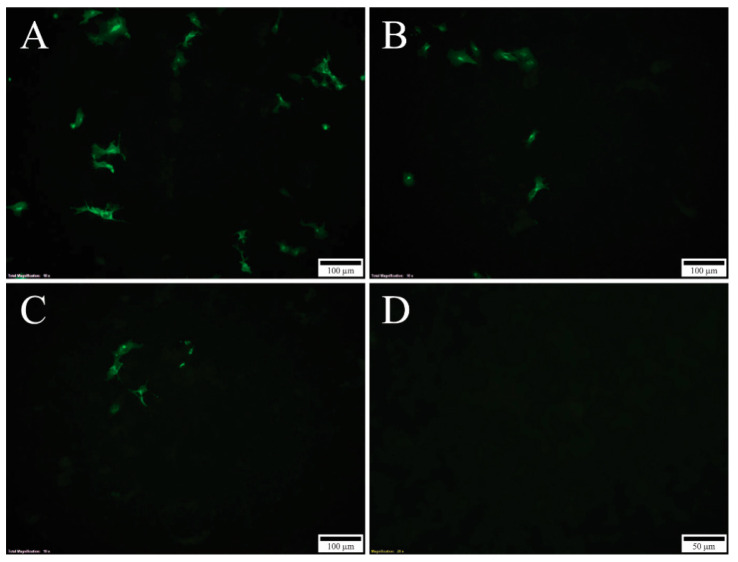
Indirect fluorescent antibody test (IFAT) using mAB targeting E2 in transfected Chinook salmon embryo cells (CHSE) cells: 4 dpt (10X). (**A**) rSAV3, (**B**) rSAV3 E2_319G_, (**C**) rSAV3 E2_319A_., (**D**) negative control. No staining was observed in the cells transfected with constructs containing E1_35_ mutations (not shown).

**Figure 4:**
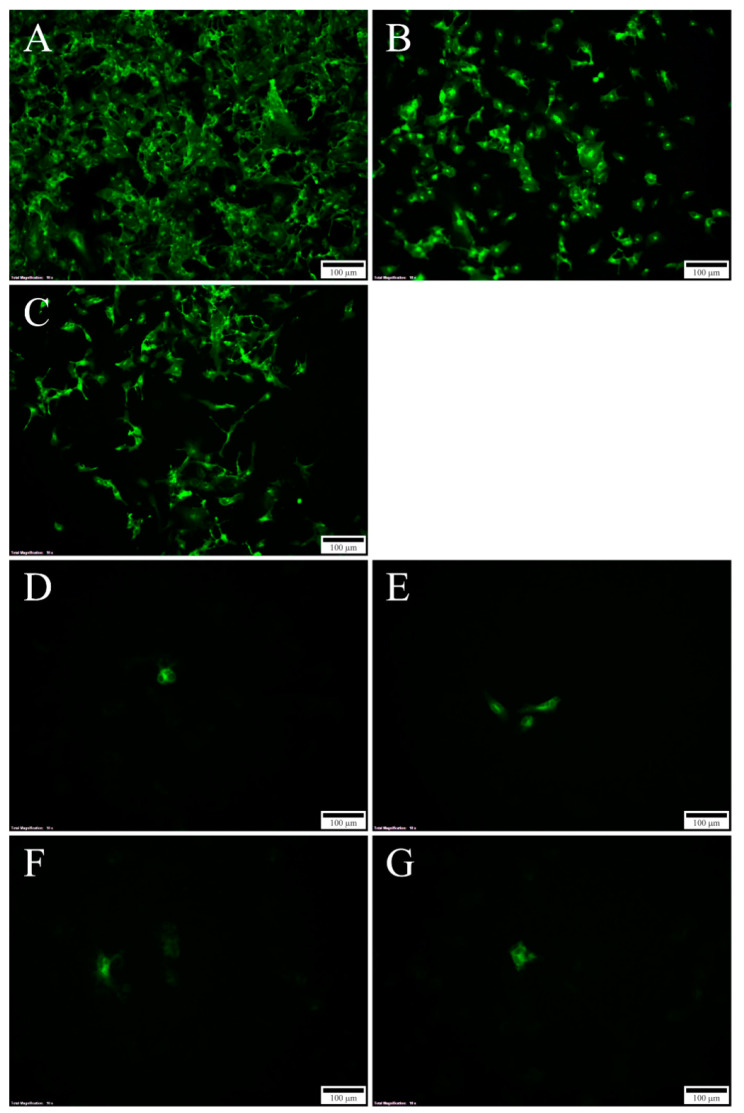
IFAT targeting E2 in CHSE-214 cells: 7 dpt, 10X; (**A**) rSAV3; (**B**) rSAV3 E2_319Q_; (**C**) rSAV3 E2_319A_; (**D**) rSAV3 E1_35Q_; (**E**) rSAV3 E1_35A_; (**F**) rSAV3 E1_35Q_ E2_319Q; and_ (**G**) rSAV3 E1_35A_ E2_319A_. Strong staining in cells transfected with the rSAV3 clone and the two constructs containing mutations in E2_319_ only (**A**–**C**). Weak or no staining in cells transfected with constructs containing mutations in E1_35_ (**D**–**G**).

Mutational changes of N-glycosylation sites were achieved by the substitution of the Asn in the motif, Asn-X-Ser/Thr, with either Gln or Ala in the infectious SAV3 clone. Gln is chemically similar to Asn, implying that the tertiary protein structure will be minimally affected by this mutation. Ala, which has a short side chain, was used as an alternative substitution to Gln. The seven generated plasmid constructs containing recombinant SAV3 sequences (Table 1) were transfected into CHSE-214 cells and the production of viral proteins was verified by the IFAT targeting the E2 protein. At 4 days post transfection (dpt), cytoplasmic staining could only be observed in cells transfected with rSAV3, rSAV3 E2_319Q_ and rSAV3 E2_319A_ (Figure 3). At 7 dpt, the number of stained cells transfected with these three constructs increased significantly (Figure 4A–C). In contrast, no staining could be observed in 4 dpt in cells transfected with the four constructs harboring mutations in E1_35_, i.e., prSAV3-E1_35Q_, prSAV3-E1_35A_, prSAV3-E1_35Q_E2_319Q_ and prSAV3 E1_35A_E2_319Q_ (not shown). However, at 7 dpi, a few positive cells could also be detected from the constructs only mutated in E1_35_ (Figure 4D–G). In mock treated cells, no staining was observed (not shown).

### 3.3. Presence of Viral RNA in Culture Medium after Passage in CHH-1 Cells

**Figure 5:**
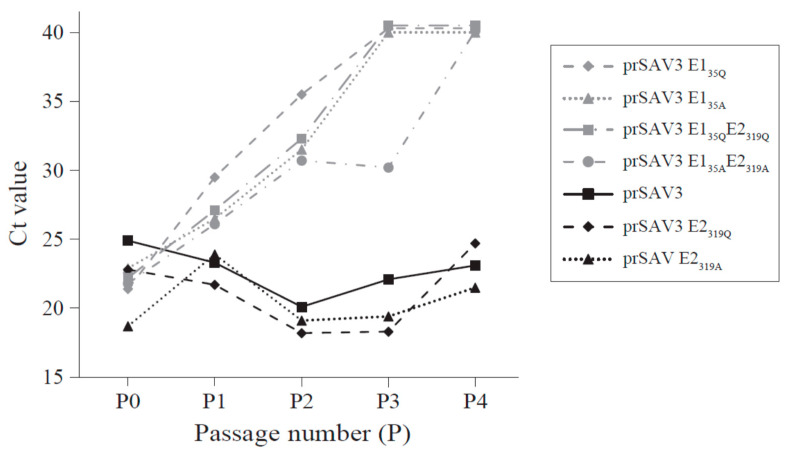
Recovery of the viral RNA in Chum salmon heart-1 cells (CHH-1) supernatant after infection with SAV3 with a mutation in the N-glycosylation sites in E1 and E2. The dots represent Ct values from RT-qPCR run with reverse transcriptase and thus both viral RNA and carryover plasmid DNA. The Ct values of rSAV3, rSAV3 E2_319A_ and rSAV3 E2_319Q_ (black dots) constructs decrease for each passage, indicating an increasing viral RNA concentration in the cell culture medium. Ct value for rSAV3 constructs containing mutations in E1 (grey dots), i.e., prSAV3 E1_35Q,_ prSAV3 E1_35A,_ prSAV3 E1_35Q/_E2_319Q,_ and prSAV3 E1_35A/_E2_319A_ increase with each passage and no Ct is observed by P4.

The presence of viral RNA in the cell culture medium was tested by RT-PCR for passage P1–P4 and cDNA synthesis was run with or without reverse transcriptase (RT) to distinguish the leftover plasmid DNA from the transfections, from viral RNA. The presence of the mutations was verified by sequencing. In samples run without RT, the Ct value increased with every passage and no Ct (i.e., threshold at 40) was obtained from cell culture media from the P4 samples (not shown). Including the RT in the RT-qPCR of the virus supernatant from prSAV3, prSAV3 E2_319Q_ and prSAV3 E2_319A_ produced overall decreasing Ct values with every passage, indicating active viral replication (Figure 5, Table S3). In contrast, the cell culture supernatant from the cells transfected with plasmids carrying the E1_35_ mutation, i.e., prSAV3 E1_35Q_, prSAV3 E1_35A_, prSAV3 E1_35Q_E2_319Q_ and prSAV3 E1_35A_E2_319A_, displayed increasing Ct values with no Ct observed from P4 for all samples. Still, the Ct values for these latter samples were always lower compared to when the RT enzyme was left out. The experiments for these four constructs were repeated to verify the results. Hence, some low-level presence of viral RNA in the cell culture medium cannot be excluded. Sanger sequencing performed on RNA isolated from P4 for rSAV3 E2_319Q_ and rSAV3 E2_319A_ confirmed that the mutations were intact after passage four in the cell culture.

### 3.4. Cytopathic Effects

**Figure 6:**
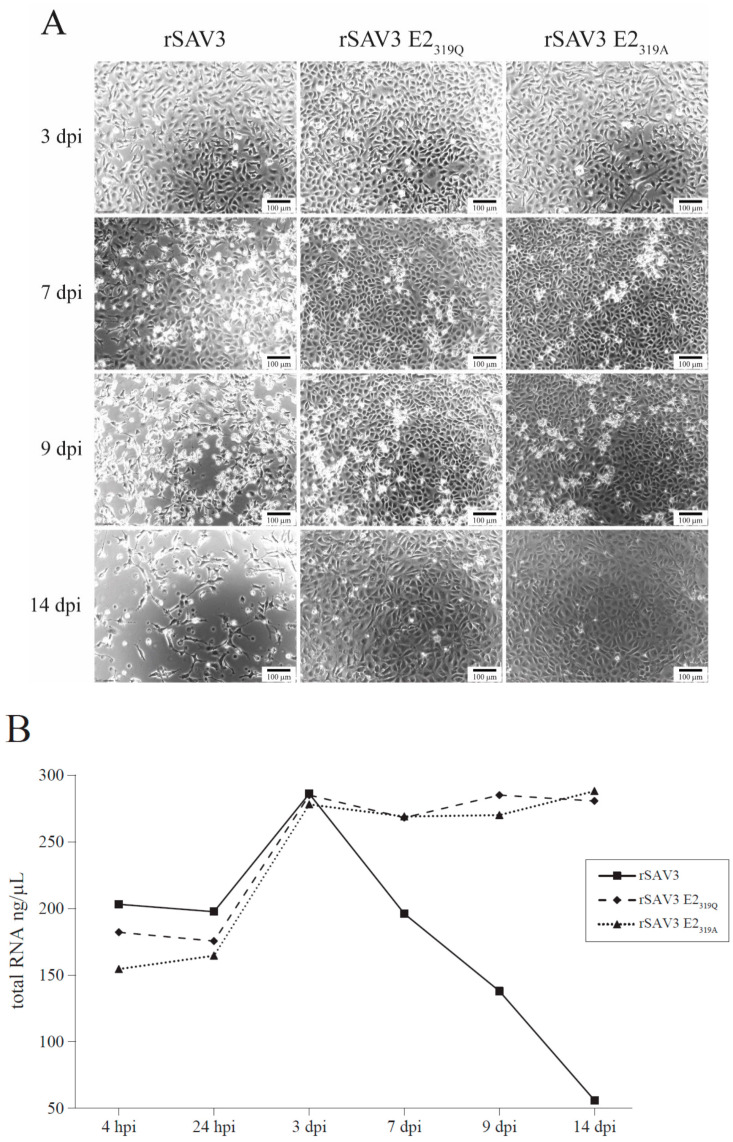
Cytopathic effect (cpe) in CHH-1 cells infected with rSAV3, rSAV3 E2_319Q_ and rSAV3 E2_319A_. (**A**) Cytopathic effect monitored by phase contrast microscopy at 3 dpi: no cpe; 7 dpi: subtle differences in wells, more rounded cells are observed for rSAV3; 9 dpi: detached cells are observed in all wells, but to a larger degree in cells infected with rSAV3 where the monolayer is partially disrupted; 14 dpi: the monolayer is completely disrupted for rSAV3, while intact for the cells infected with rSAV3 E2_319_ mutants. (**B**) The total RNA (ng/µL) in adherent cells. All wells have an increase in RNA concentration until day 3 pi. The amount of cellular RNA in rSAV3 E2_319_ mutants infected cells stabilizes thereafter, while that of rSAV3-infected cells rapidly decrease.

In cells infected with SAV, the cpe in CHSE cells is usually characterized as elongated, vacuolated cells with pseudopodia-like extensions and curled up dead cells [29]. No cpe was observed in CHH cells for either non-mutated or mutated virus variants at 3 dpi (Figure 6A). From 7 dpi, detached dead cells were seen in the media. Even though the differences were subtle, the cell layers infected with rSAV3 showed more rounded cells at this timepoint (Figure 6A). At 9 dpi, the detached dead cells were observed in all wells, but the cpe was more pronounced in the wells infected with rSAV3, where the monolayer was partially disrupted (Figure 6A). The monolayer was completely disrupted for rSAV3 at 14 dpi, while the monolayers were recovering and partly intact with cells infected with rSAV3 E2_319Q_ and rSAV3 E2_319A_ at this timepoint (Figure 6A).

The total RNA from the attached cells in the wells were quantified to obtain a measure of the ratio of detached cells due to virus infection. The same number of cells was added to each well at the onset of the experiment. The results showed that for the cells infected with rSAV3, the total RNA concentration peaked at 3 dpi (~300 ng/µL), while at 7 dpi it had declined substantially and this decline continued until the end of the experiment at 14 dpi, when the total RNA was below 50 ng/µL (Figure 6B, Table S4). For rSAV3 E2_319Q_ and rSAV3 E2_319A_ on the other hand, the total RNA increased until 7 dpi and stayed above 250 ng/µL until the end of the experiment at 14 dpi (Figure 6B, Table S4).

### 3.5. Quantification of Intracellular Viral RNA from Adherent CHH-1 Cells

**Figure 7:**
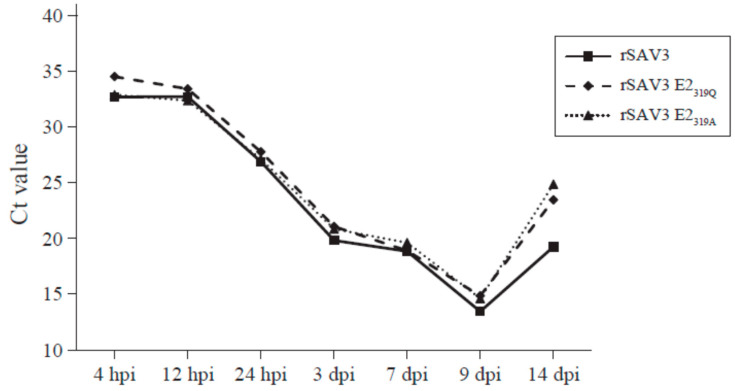
Viral Ct values of CHH-1 cells infected with rSAV3, rSAV3 E2_319Q_ and rSAV3 E2_319A_.

In order to investigate whether differences in viral RNA levels could be observed between the rSAV3 and the two mutants rSAV3 E2_319Q_ and rSAV3 E2_319A_, RT-qPCR was performed on infected, adherent CHH-1 cells. Viral RNA was detectable for all three virus variants from 4 hpi (Figure 7, Table S5). A decrease in the viral Ct comparable for all three virus variants occurred from 24 hpi to 3 dpi, i.e., before the cpe was observed. The decrease in viral Ct continued until 9 dpi when it peaked. At 14 dpi, viral RNA levels decreased for all three variants, but the drop was higher for the two mutated rSAV3 E2_319Q_ and rSAV3 E2_319A_ (Figure 7, Table S5).

Between 4 hpi and 9 dpi, the viral RNA levels were comparable for all three virus variants. The Ct values were average values from two biological and two qPCR parallels for each sample, except for 24 hpi, where only one biological sample was used (Ct values are shown in Table S5).

### 3.6. Titer of Infectious Virus Produced by CHH-1 Cells

**Figure 8:**
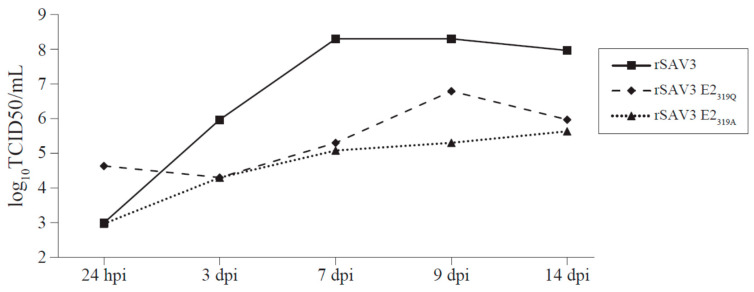
Tissue culture infective dose (TCID50/mL) of rSAV3, rSAV3 E2_319Q_ and rSAV3 E2_319A_ mutants in media from the infected CHH cells.

Quantification of the infectious virus from CHH-1 cells infected with rSAV3, rSAV3 E2_319Q_ and rSAV3 E2_319A_ was performed (Figure 8). Ten-fold more infectious viruses were present in the supernatant 24 hpi for rSAV3 E2_319Q_ compared to rSAV3 and rSAV3 E2_319A._ From 24 hpi to 3 dpi, however, the virus titer from rSAV3 increased almost 1000-fold, from 9.9 × 10^2^ to 9.3 × 10^5^. However, at 3 dpi, the rSAV3 infectious viral particles in supernatant had almost increased 1000-fold, from 9.9 × 10^2^ to 9.3 × 10^5^ TCID50/mL. In comparison, rSAV3 E2_319Q_ and rSAV3 E2_319A_ had titers of 2.0 × 10^4^ TCID50/mL at 3 dpi. The rSAV3 E2_319A_ titer increased only to 4.3 × 10^5^ TCID50/mL at 14 dpi, while rSAV3 E2_319Q_ increased to 6.2 × 10^6^ at 9 dpi_,_ and then dropped to 9.3 × 10^5^ at 14 dpi. The TCID50/mL for rSAV3 increases peaked at 2 × 10^8^ on 7 dpi and was 9.3 × 10^7^ TCID50/mL at 14 dpi. The difference of infectious virus in the medium between the non-mutated rSAV3 and the mutated rSAV3 E2_319_ variants was in the range of 10^2^–10^3^ TCID50/mL from 7 dpi and onwards.

## 4. Discussion

In the present work, we studied the effects of the site-directed mutation of glycosylation sites in the SAV3 envelope proteins, E1 and E2, regarding virus replication in cell culture. Glycans on viral envelope proteins are associated with viral infectivity and fitness, protein folding, assembly and immune recognition and evasion [41,42].

Sequence alignments with other alphaviruses showed that the location of predicted N-glycosylation sites in the SAV3 glycoproteins are not well conserved at the primary sequence level. However, the 3D structures modeled for SAV3 E1 and E2 revealed similar spatial positioning of these two sites to that of other alphaviruses. VEEV E1_134N_ locates to the middle region of a β-strand on the surface of the protein [40] and in our model, the SAV3 E1_35N_ also located to the surface region in the center of a predicted β-strand. The E1 subdomain I harboring the glycosylation [35,39] is conserved in both alphavirus proteins. The N-glycosylation site in SAV3 E2_319N_ were, as for VEEV E2_318N_ [40], predicted to be buried near the lipid membrane. The SAV3 E2_319N_ was located within a predicted coil region and VEEV E2_318N_ in the center of a β-strand, both residing within a conserved structural fold. Altogether, this provides strong support that SAV3 E1_35N_ and SAV3 E2_319N_ are functional N-glycosylation sites.

The glycoproteins embedded in the envelope of the alphaviruses affect viral infectivity, viral particle formation and immune evasion [43]. Individual N-linked glycosylations has different importance, some can be eliminated with little consequences while others appear to be essential [9]. The glycosylation pattern depends on the host glycosylation machinery, a particularly important aspect of alphaviruses of terrestrial vertebrates since they replicate in both an invertebrate vector and a vertebrate host, where the glycans in the former are distinguishable from those synthesized in the latter by the absence of complex-and hybrid-type N-linked oligosaccharides [17]. For RRV, the lower complexity of N-linked glycans on virions derived from insect cells have been shown to be linked to a higher efficiency of infection due to the reduced ability to induce type I IFN in mammalian cells compared to the virus grown in mammalian cells [44,45]. SAV, on the other hand, is not transmitted by arthropod vectors and in a farming situation, it does not transit between different host species. Mammalian alphaviruses often have two N-linked glycosylation sites in E2, whereas SAV3 only has one. Whether this difference is associated with the lack of dependency on an arthropod vector cannot be excluded.

We recovered the infectious virus for the E2_319_ mutants only, i.e., prSAV3 E2_319A_ and E2_319Q_. In the IFAT of the E2_319_ mutants, a modest number of stained cells was observed at 4 dpt, which increased to most cells in the monolayer at 7 dpt, when little differences were seen compared to the parental strain, rSAV3. The staining pattern indicated that the spread to these cells could be due to a virus shed in the supernatant.

For the four clones mutated in E1_35_, both those only muted in E1 and those with combined mutations in E1 and E2, very limited staining could be observed from IFAT. There was no spread to neighboring cells, virus RNA could not be detected following multiple passaging as determined by RT-qPCR on cell culture supernatant and in conclusion, no infectious virus was recovered for the E1_35_ mutants.

The rSAV3 E2_319Q_ and rSAV3 E2_319A_ infected CHH-1 cells produced virus particles that were shed to the culture medium. This could be measured as viral RNA or as infectious particles in the cell medium. The infectious virus titer for rSAV3, measured by TCID_50_/mL, was 10^2^–10^3^ higher than those of rSAV3 E2_319G_ and rSAV3 E2_319A_. Furthermore, the ability to cause cpe also differed between the rSAV3 and the rSAV3 E2_319_ mutants. At 9 dpi, the monolayer was disrupted for rSAV3-infected cells, and at 14 dpi the monolayer was completely lost, while the monolayers were intact for the rSAV3 E2_319_ variants. In accordance with this, the total RNA of the attached cells was significantly lower in CHH-1 cultures from 7 dpi for the rSAV3 compared to the rSAV3 E2_319_ variants. A less infectious virus was produced and less cpe occurred for E2_319A_ and E2_319Q_ in the CHH-1 cells, indicating that the rSAV3 E2_319_ variants were attenuated in cell culture compared to the parental rSAV3.

All three E2_319_ variants displayed comparable amounts of viral RNA in CHH-1 cells 4-12 hpi suggesting that the cellular uptake of the mutants is not influenced by the lack of glycan in E2_319_. In a study of SINV, the absence of either of its two E2 N-glycosylation sites increased replication and virulence in mammalian cells, thought to be due to an increase in the efficiency of binding to heparan sulphate (HS) [20]. One of the two SINV E2 glycosylation sites, N_318_, is located in the center of a β-strand [32], which when superimposed is analogous to the N_318_ site in VEEV E2 (not shown) and hence the N_319_ site in SAV3. Thus, even if the glycosylation site in SAV3 E2_319_ is predicted to be somewhat buried in the protein near the outer lipid membrane, influence on receptor binding cannot be excluded. Binding to cell surface HS can be the initial attachment factor for a virus particle and the first step for viral entry into the cell [46]. For SINV, RRV, and VEEV, a single amino acid substitution caused increased affinity for HS, believed to be a result of cell culture adaptation [47,48,49]. A similar single amino acid substitution after passages in cell culture was been observed for SAV3 indicating that the increased affinity of HS can affect viral fitness in cell culture [50]. It should not be excluded that possible differences in viral RNA levels at early timepoints, although not consistent for rSAV3, can be associated with an increase in affinity to HS or similar attachment factor for the SAV3 E2319 mutants. 

At 3 dpi, the amount of viral RNA in the infected CHH-1 cells was at the same level for the E2_319_ mutants and the parental rSAV3. A similar observation was made at 7 dpi, but at this time it was 1000-fold more infectious virus particles in the cell culture medium of the rSAV3-infected cells than in the cells infected with the E2_319_ mutants. This indicates that the qPCR and Ct values of cells are not suited for the assessment of the amount of produced infectious SAV. The lower shedding of virus from the two E2_319_ single mutants can therefore not be explained by differences in the virus uptake or lower levels of viral RNA synthesis, but may indicate that the differences are linked to lower efficiency in generating the E1–E2 heterotrimer in the absence of glycan at E2_319N_. Together with the single amino acid substitution introduced, this may affect proper spike assembly. The surface expression of SAV3 E2 occurs only in the presence of E1, and in the absence of E1, E2 (p62) is arrested in the endoplasmic reticulum (ER) [11].

Substitution in the N-linked glycosylation site of E1_35_ abolished the recovery of the infectious virus after transfection, and further passages of the supernatants in cell culture did not enable the recovery of the virus. The transfection was evaluated by the IFAT of CHSE cells at 4, 7 and 10 dpt, using a MAb recognizing E2. Transfection with prSAV3 E1_35_ gave from none to very few stained cells, and there was no further spread to neighboring cells, and the additional mutation of E2_319_, i.e., transfection with prSAV3 E1_35**/**_E2_319_ did not compensate this. In general, the deglycosylation by glucosidase inhibitors or the mutagenesis of the glycosylation sites may cause misfolding and the retention of viral glycoproteins within the ER [51]. With the documented reciprocal dependency of E1 and E2 in generating functional spikes, the lack of expression of E2 in cells when E1 was altered was therefore not unexpected. During SAV infection, the capsid is cleaved off the structural polyprotein, and the remaining p62-6K-E1 translocates to the ER, where E1 and E2 undergo post-translational modifications [12]. The lack of the glycan at E1-N_35_ could therefore interfere with the processing of p62-6K-E1 in the ER. The staining of prSAV3 E1_35_ in a few cells using a monoclonal antibody targeting E2 indicated that in these particular cells, the E2 protein, or its precursor p62, was expressed and E1–E2 spikes were made, and the interference with production of infectious particles occurred after the synthesis of the E1–E2 spikes. There were no differences in the Ct values of RT-qPCRs run with or without a RT step from supernatants of the prSAV3 E1_35_ transfected cells and of subsequent passages. This indicated that viral RNA was not present in the supernatants of prSAV3 E1_35_ transfected cells nor in subsequent passages, and thus indicated a lack of shed virus particles. An additional mutation in E2, i.e., the double E1_35_/E2_319_ mutants, did not compensate for the lack of recovery. The glycosylation site in E1 plays a major role in the pathogenesis of SAV3, and the removal of this site rendered the virus not viable. For SINV, the deletion of either of its two N-glycosylation sites in E1 did not result in complete inactivation but decreased the replication in Baby hamster kidney (BHK) cells and virulence in mice [20]. In addition, for SINV, a potential influence on the fusion process mediated by E1 was associated with N-glycosylation, as well as the partial restorage of membrane fusion activity in a double mutant defective in one of the E2 N-glycosylation sites [21].

## 5. Conclusions

In conclusion, the mutations of the glycosylation sites of the SAV3 envelope proteins severely altered the virulence and production of an infectious virus in cell culture. Substitution in the N-linked glycosylation site in E2 attenuated the virus in cell culture, whereas the mutation of the N-glycosylation motif in E1 resulted in complete viral inactivation. The glycans were predicted to be important for the structure and function of the envelope proteins and can potentially be a useful strategy in further functional studies. SAV variants defective in the E2 N-glycosylation site may, preferably, combined with targeted mutations in other regions of the viral genome to reduce the risk of reversion to virulence, be employed to generate robust live attenuated vaccines.

## Figures and Tables

**Table 1:** Expression plasmids.

Plasmid Constructs	Mutation(s)
prSAV3	-
prSAV3 E1_35Q_	E1_35N→Q_
prSAV3 E1_35A_	E1_35N→A_
prSAV E2_319Q_	E2_319N→Q_
prSAV E2_319A_	E2_319N→A_
prSAV3 E13_5Q_ E2_319Q_	E1_35N→Q_/E2_319N→Q_
prSAV3 E1_35A_ E2_319A_	E1_35N→A_/E2_319N→A_

## Supplementary Materials

The following are available online at https://www.mdpi.com/1999-4915/12/10/1071/s1, Figure S1: Multiple sequence alignments of alphavirus E1 and E2 protein sequences, Figure S2: PSIPRED secondary structure predictions of E1 and E2 from SAV3 and Venezuelan equine encephalitis virus and Sinbid virus, Table S1: Pairwise amino acid sequence identities and similarities between E1 from SAV3 and selected alphaviruses, Table S2: Pairwise amino acid sequence identities and similarities between E2 from SAV3 and selected alphaviruses, Table S3. Ct values following recovery of viral RNA in CHH-1 supernatant after infection with SAV3 with mutation in N-glycosylation sites in E1 and E2, Table S4. Cytopathic effect in CHH-1 cells infected rSAV3, rSAV3 E2_319Q_ and rSAV3 E2_319A_ as measured by total RNA concentration (ng/µL) in adherent cells, Table S5. Viral Ct values of CHH-1 cells infected with rSAV3, rSAV3 E2_319Q_ and rSAV3 E2_319A._
