# Supplementary material for: Mutation of N-glycosylation Sites in Salmonid Alphavirus (SAV) Envelope Proteins Attenuate the Virus in Cell Culture
Source: Viruses. 2020 Sep 24;12(10):1071. doi: 10.3390/v12101071 (PMC7650630; doi:10.3390/v12101071)
Supplement: Supplementary file 1 [file viruses-12-01071-s001.zip › viruses-928200-supplementary.pdf]

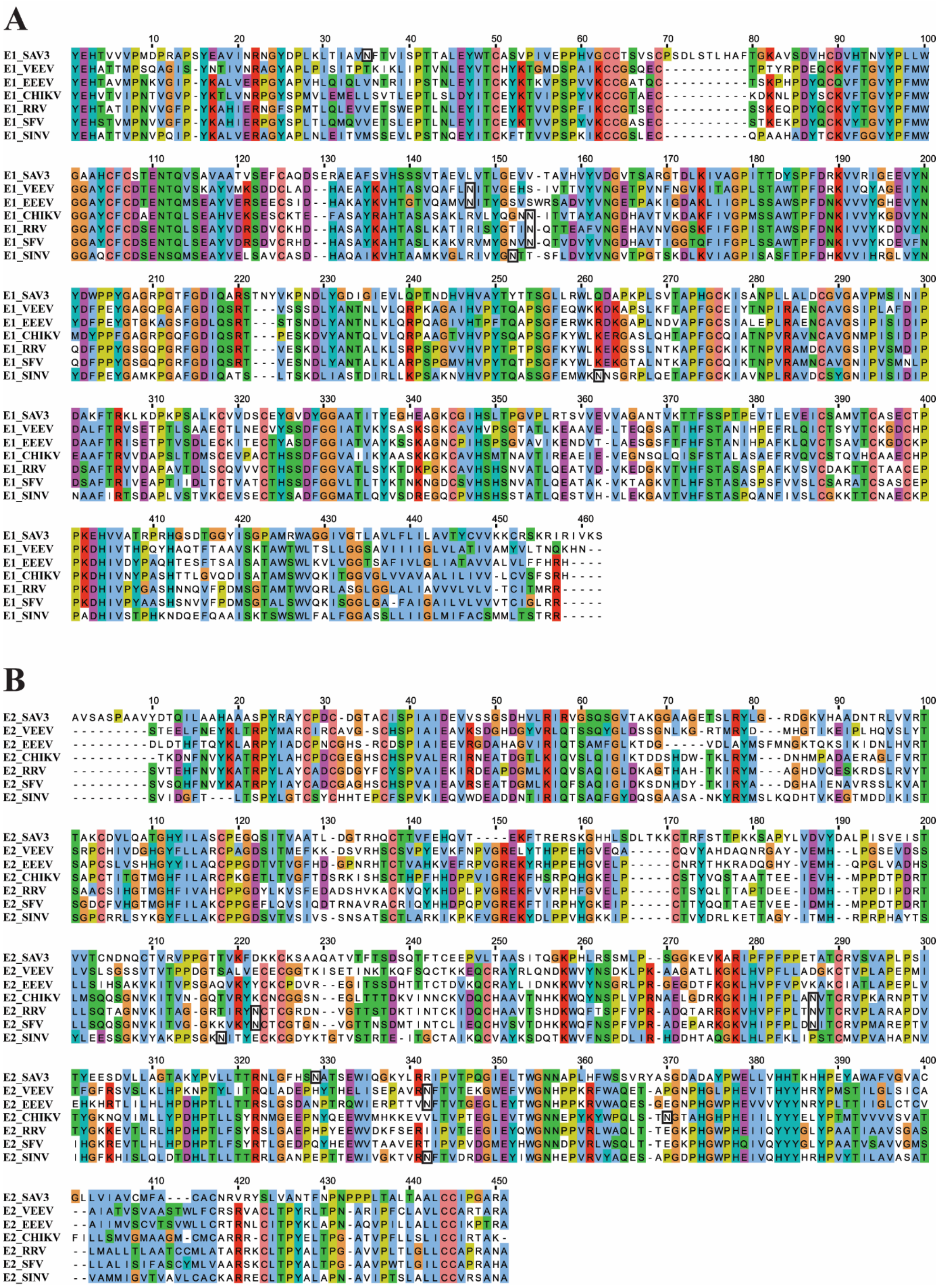

Figure S1. Multiple sequence alignments of the **A** E1 and **B**) E2 protein sequences from SAV3 and the alphaviruses Venezuelan equine encephalitis virus (VEEV) (3J0C_A/B, P09592 (like)), Eastern equine encephalitis virus (EEEV) (ANB41743), Chikungunya virus (CHIKV) (AEA10291), Ross river virus (RRV) (P08491), Semliki forest viru (SFV) (NP_819008, NP_819006) and Sinbid virus (SINV)

(CAA24684). The predicted N-glycosylation sites for SAV E1 and E2, and those from the other alphaviruses, are shown in black boxes. Dotted lines in bold indicate sequence sections removed for display purposes.

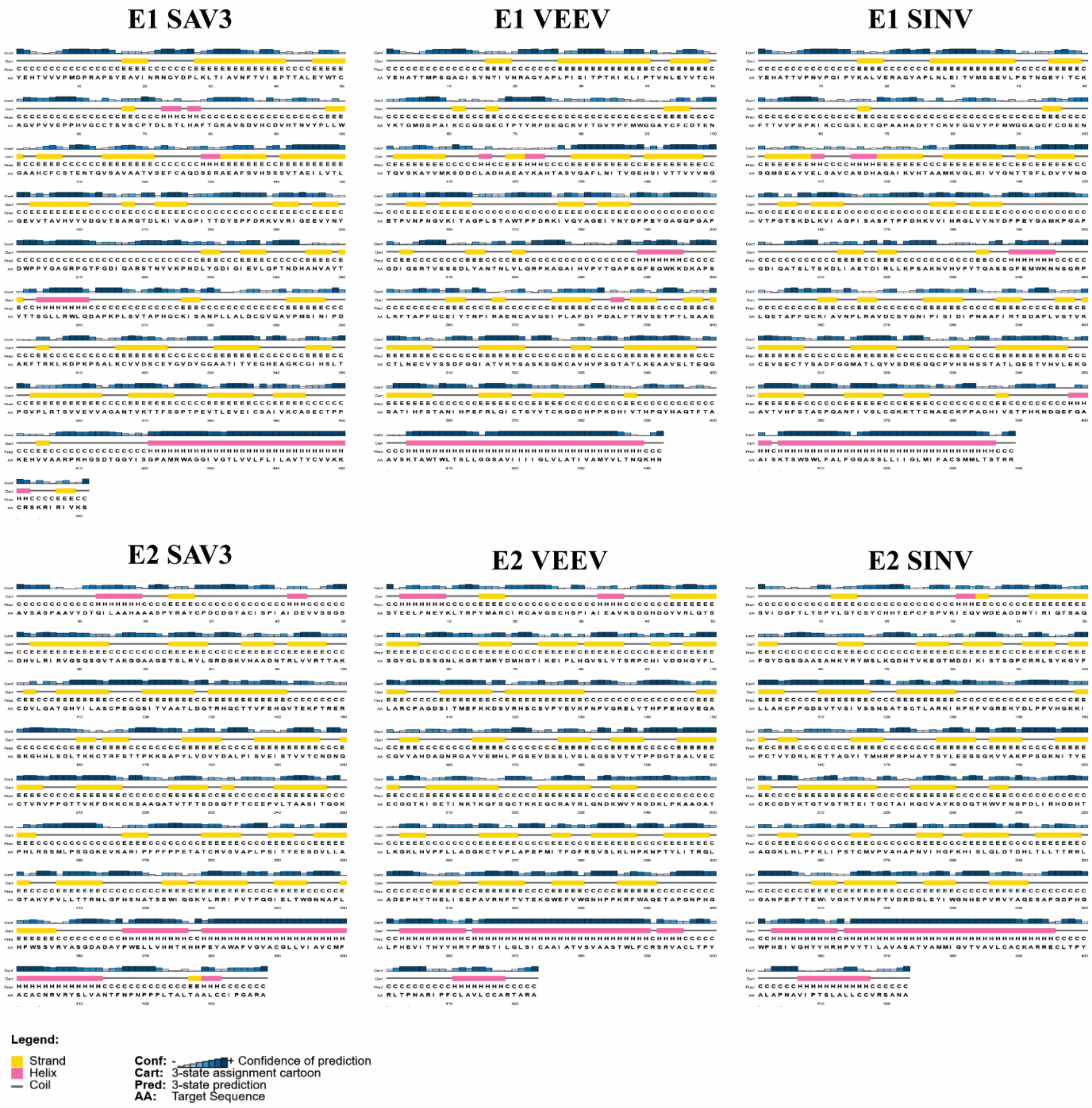

Figure S2. PSIPRED secondary structure predictions of **A** E1 and **B** E2 from SAV3 and the alphaviruses Venezuelan equine encephalitis virus (VEEV) (3J0C_A/B, P09592 and Sinbid virus (SINV) (CAA24684).

Table S1. Pairwise amino acid sequence identities (above diagonal) and similarities (below diagonal) between E1 from SAV3 and selected alphaviruses (%).

	SAV3	VEEV	EEEV	CHIKV	RRV	SFV	SINV
**SAV3**	**-**	38.2	40.0	37.4	38.8	39.7	39.4
**VEEV**	49.3	-	60.1	50.8	52.3	51.4	50.3
**EEEV**	50.8	70.5	-	51.7	54.6	56.6	52.8
**CHIKV**	49.4	64.0	65.1	-	60.5	61.2	46.7
**RRV**	50.0	62.3	66.9	71.9	-	77.9	50.9
**SFV**	49.3	60.5	67.4	71.9	86.5	-	49.3

**SINV**	50.6	60.4	62.6	57.4	59.8	58.7	-

Table S2. Pairwise amino acid sequence identities (above diagonal) and similarities (below diagonal) between E2 from SAV3 and selected alphaviruses (%).

	SAV3	VEEV	EEEV	CHIKV	RRV	SFV	SINV
**SAV3**	**-**	27.2	28.3	29.5	28.2	26.8	25.8
**VEEV**	38.3	-	48.1	36.1	38.9	36.0	40.7
**EEEV**	38.1	59.0	-	40.5	40.5	39.0	43.8
**CHIKV**	42.0	49.6	52.4	-	56.3	57.5	35.6
**RRV**	38.9	49.8	53.6	65.6	-	69.2	40.8
**SFV**	37.9	48.1	51.0	66.5	78.4	-	39.6
**SINV**	37.4	50.8	56.0	46.3	51.2	49.1	-

Table S3. Ct values following recovery of viral RNA in CHH-1 supernatant after infection with SAV3 with mutation in N-glycosylation sites in E1 and E2.

Virus	Passage number				
	P0	P1	P2	P3	P4
rSAV3 E1$_{35Q}$	21.4	29.5	35.5	no Ct	no Ct
rSAV3 E1$_{35A}$	22.9	26.5	31.5	no Ct	no Ct
rSAV3 E1$_{35Q}$ E2$_{319Q}$	22.2	27.1	32.3	no Ct	no Ct
rSAV3 E1$_{35A}$ E2$_{319A}$	21.8	26.1	30.7	30.2	no Ct
rSAV3	24.9	23.3	20.1	22.1	23.1
rSAV E2$_{319Q}$	22.8	21.7	18.2	18.3	24.7
rSAV E2$_{319A}$	18.7	23.9	19.1	19.4	21.5

Assays were repeated for constructs containing mutations in E1 to verify pattern of decreasing Ct-values with no Cts at P4. Sanger sequencing on RNA isolated from P4 confirmed that the mutations in rSAV E2$_{319Q}$ and rSAV E2$_{319A}$ were intact.

Table S4. Cytopathic effect in CHH-1 cells infected rSAV3, rSAV3 E2$_{319Q}$ and rSAV3 E2$_{319A}$ as measured by total RNA concentration (ng/μL) in adherent cells.

Virus	4 hpi		24 hpi		3 dpi		7 dpi		9 dpi		14 dpi	
	#1	#2	#1	#2	#1	#2	#1	#2	#1	#2	#1	#2
rSAV3	175	157	202	193	287	285	181	211	134	142	47	65
rSAV E2$_{319Q}$	169	165	155	196	331	284	326	210	322	248	256	305
rSAV E2$_{319A}$	153	184	143	186	278	263	326	212	232	308	267	309

Biological parallels (well #) for each virus at each timepoint. hpi=hours post infection, dpi= days post infection. Total RNA concentration in ng/μL.

Table S5. Viral Ct values of CHH-1 cells infected with rSAV3, rSAV3 E2$_{319Q}$ and rSAV3 E2$_{319A}$

Virus	4 hpi		12 hpi		24 hpi		3 dpi		7 dpi		9 dpi		14 dpi	
	#1	#2	#1	#2	#1	#2	#1	#2	#1	#2	#1	#2	#1	#2
rSAV3	32.7	33.0	31.7	33.6	*no Ct*	*no Ct*	19.3	20.5	19.8	17.8	*29.4*	13.4	18.8	20.2
	32.2	32.8	31.8	33.7	*no Ct*	27.6	19.2	20.3	19.8	18.0	*29.8*	13.5	18.2	19.8
	-	-	-	-	*no Ct*	27.4	-	-	-	-	-	-	-	-
	-	-	-	-	*no Ct*	27.7	-	-	-	-	-	-	-	-
	-	-	-	-	*no Ct*	25.9	-	-	-	-	-	-	-	-
	-	-	-	-	*no Ct*	25.8	-	-	-	-	-	-	-	-
rSAV E2$_{319Q}$	35.0	33.4	33.0	34.0	27.9	27.4	21.7	20.5	18.4	19.5	15.2	14.5	22.7	24.2
	35.9	33.7	33.0	33.6	27.9	27.8	21.5	20.6	18.4	19.3	15.0	14.7	22.7	24.2
rSAV E2$_{319A}$	33.0	32.8	31.9	32.8	27.4	26.9	21.5	20.4	20.6	18.7	13.3	16.1	24.7	24.9
	33.0	32.7	31.7	33.0	27.1	27.0	21.4	20.1	20.5	18.7	12.9	16.2	24.8	25.0

Biological (well #) and PCR parallels for each virus at each timepoint. hpi=hours post infection, dpi= days post infection. Data in italics have not been included in Figure. 7. See main text for more details.
